# Adaptation to Overflow Metabolism by Mutations That Impair tRNA Modification in Experimentally Evolved Bacteria

**DOI:** 10.1128/mbio.00287-23

**Published:** 2023-02-28

**Authors:** Marc J. Muraski, Emil M. Nilsson, Melissa J. Fritz, Anthony R. Richardson, Rebecca W. Alexander, Vaughn S. Cooper

**Affiliations:** a Department of Microbiology and Molecular Genetics and Center for Evolutionary Biology and Medicine, University of Pittsburgh, Pittsburgh, Pennsylvania, USA; b Department of Chemistry, Wake Forest University, Winston-Salem, North Carolina, USA; Pennsylvania State University

**Keywords:** experimental evolution, tRNA modification, tradeoff, translational control

## Abstract

When microbes grow in foreign nutritional environments, selection may enrich mutations in unexpected pathways connecting growth and homeostasis. An evolution experiment designed to identify beneficial mutations in Burkholderia cenocepacia captured six independent nonsynonymous substitutions in the essential gene *tilS*, which modifies tRNA^Ile2^ by adding a lysine to the anticodon for faithful AUA recognition. Further, five additional mutants acquired mutations in tRNA^Ile2^, which strongly suggests that disrupting the TilS-tRNA^Ile2^ interaction was subject to strong positive selection. Mutated TilS incurred greatly reduced enzymatic function but retained capacity for tRNA^Ile2^ binding. However, both mutant sets outcompeted the wild type by decreasing the lag phase duration by ~3.5 h. We hypothesized that lysine demand could underlie fitness in the experimental conditions. As predicted, supplemental lysine complemented the ancestral fitness deficit, but so did the additions of several other amino acids. Mutant fitness advantages were also specific to rapid growth on galactose using oxidative overflow metabolism that generates redox imbalance, not resources favoring more balanced metabolism. Remarkably, 13 *tilS* mutations also evolved in the long-term evolution experiment with Escherichia coli, including four fixed mutations. These results suggest that TilS or unknown binding partners contribute to improved growth under conditions of rapid sugar oxidation at the predicted expense of translational accuracy.

## INTRODUCTION

Evolution experiments coupled with whole-genome sequencing (WGS) harness the nearly deterministic effect of selection in large populations to capture beneficial mutants (1). Any mutation that rises quickly to high frequency (e.g., within 50 to 500 generations) is essentially guaranteed to be adaptive or part of an adaptive genotype (2, 3). Even though beneficial mutations are relatively rare, in bacterial populations numbering 10^7^ cells or more, numerous beneficial mutants often arise and co-occur. Under these cases, mutations of smaller benefit are likely lost because they are outcompeted by co-occurring mutants of greater benefit, which causes mutants captured first to be among the most beneficial. Surprisingly, these experiments have not captured the diversity of genetic targets that one might anticipate from a genome-wide screen of beneficial variation. Rather, experimental evolution of replicate microbial populations has revealed surprising parallelism at the gene level for early adaptations (4–6). More surprising, the identity of repeatedly mutated genes is often unexpected, and they may contribute to pathways unanticipated to play an essential role in microbial fitness. For example, in the famous long-term evolution experiment (LTEE) led by Richard Lenski in which Escherichia coli populations were grown in a simple glucose solution for >70,000 generations (7), the first few beneficial mutations that swept to fixation in each population included a deletion of genes involved in ribose catabolism and mutations in a regulator of the stringent response, *spoT* (8, 9). Neither of these gene targets were predicted causes of adaptation *a priori*, and 20 years after their discovery, exactly how these mutations are adaptive remains incompletely understood. These and other mutants, however, have inspired many subsequent studies that have improved models of how E. coli metabolism is regulated for optimal growth (10).

Previously, we developed a marker-deflection assay (11, 12) to capture the first beneficial mutations rising to high frequency in experimentally evolving populations of the generalist environmental bacterium Burkholderia cenocepacia (13). This project was motivated by the goal of comparing the spectrum of captured beneficial mutations in a metabolically versatile microbe, such as B. cenocepacia, to those identified in a domesticated laboratory strain of E. coli using similar methods (14). We hypothesized that a broader range of adaptations would be captured in the environmental microbe than in the laboratory strain, but our results quickly rejected this hypothesis and led us to focus on the highly unusual beneficial mutations identified by WGS.

We report strong positive selection on six, nonsynonymous single nucleotide substitutions in the *tilS* gene in B. cenocepacia (strain HI2424). This gene encodes tRNA^Ile^ lysidine synthetase (TilS) and is considered essential, meaning that deletion mutants are inviable (15–17). TilS modifies the tRNA isoleucine acceptor (tRNA^Ile2^) to decode the minor isoleucine codon AUA (18). Most bacterial species encode a tRNA^Ile2^ with a CAU anticodon, which it shares with tRNA^Met^, instead of a Watson-Crick complementary UAU anticodon. To avoid mistranslation, posttranscriptional modification by TilS converts the tRNA^Ile2^ wobble nucleotide C34 to lysidine (L) by adding lysine to this cytosine, which shifts base pairing specificity (15). Similarly, the presence of lysidine in tRNA^Ile2^ shifts aminoacyl-tRNA synthetase preference from methionyl-tRNA synthetase to isoleucyl-tRNA synthetase (15, 19). Because enzymatic modification of tRNA^Ile2^ is essential to maintain translational fidelity, the discovery of multiple mutations in *tilS* associated with adaptations in laboratory culture is altogether unexpected.

Further, we identified five additional mutants from these same evolution experiments in the tRNA^Ile2^ that is modified by TilS. Together, these mutants imply strong selection to alter the interaction between TilS and tRNA^Ile2^ to improve B. cenocepacia fitness in a defined laboratory medium. Here, we propose and evaluate possible hypotheses for these results that could connect translational fidelity to improved growth and metabolism with galactose as sole carbon source. We discovered that rapid growth in a galactose minimal medium (GMM) with insufficient buffering capacity caused overflow metabolism, acidification, and redox imbalance, in which *tilS* and tRNA^ile2^ mutants were favored. These studies demonstrate new connections between central metabolism and translational fidelity in which bacteria may sacrifice protein accuracy by altering essential gene products when metabolic constraints demand.

## RESULTS

### Strong genetic parallelism among adaptive mutations.

**FIG 1:**
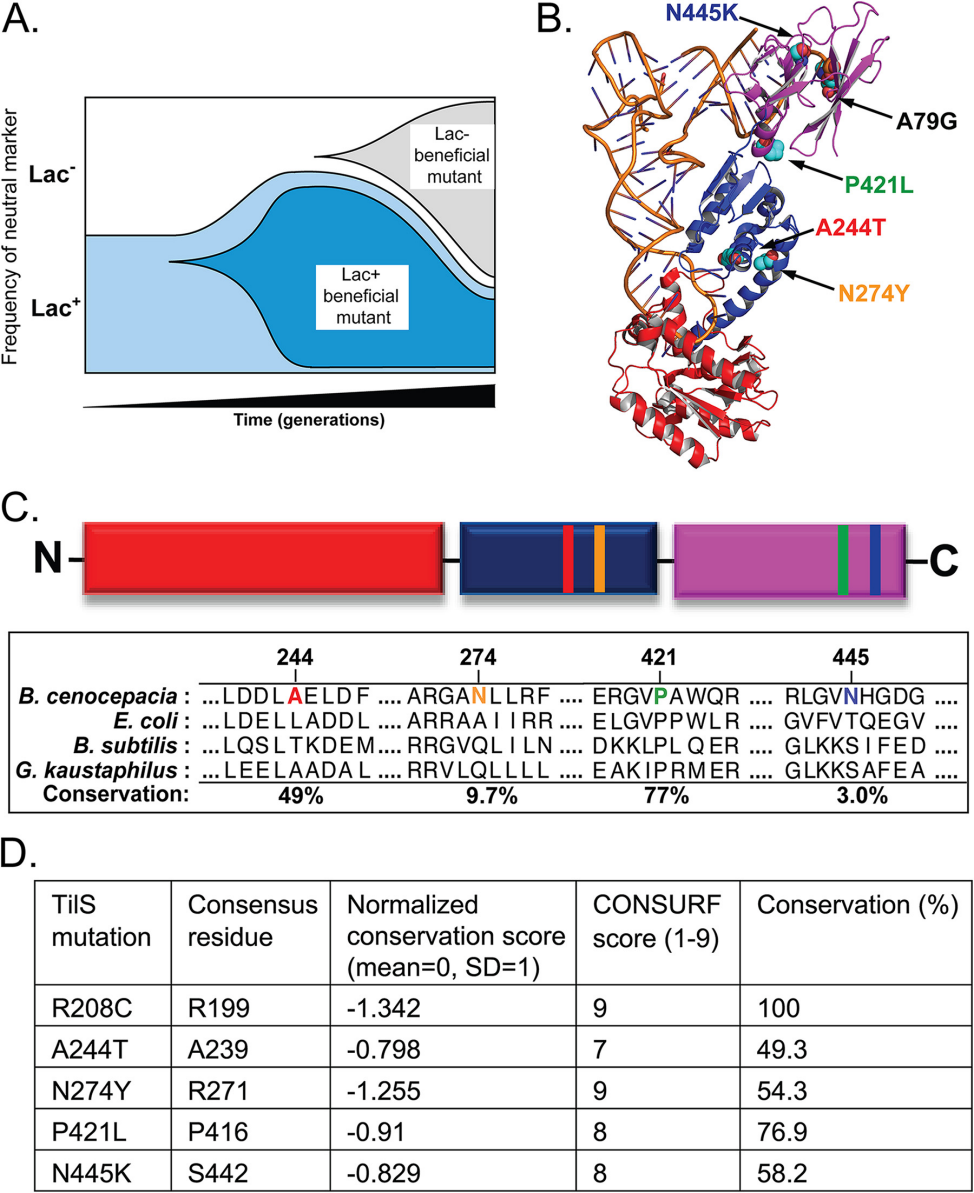
Identification of B. cenocepacia TilS and tRNA^Ile2^ mutants that increased fitness in minimal medium containing 1% galactose. (A) Schematic of marker deflection assay, where beneficial mutations can arise in either the Lac^+^ or the Lac^–^ ancestor population and affect the frequency of the marker sufficiently to indicate mutant invasion. (B) Structure of TilS:tRNA^Ile2^ complex indicating the location of single nucleotide substitutions in both partners at locations distal from the enzyme active site. Red, catalytic domain; blue, linker domain; purple, acceptor stem binding domain. Shown is the *G. kaustophilus* TilS:B. subtilis tRNA^Ile2^ structure, PDB 3A2K (22). (C) Schematic of TilS domain organization with mutation locations noted using the same color scheme as panel B, and degree of conservation of mutated residues among 150 phylogenetically diverse orthologs ranging from 35 to 95% overall identity, measured using CONSURF (23). A static CONSURF analysis of TilS can be found for PDB 3A2K on the web server. (D) Evolutionary conservation of mutated TilS residues. Normalized conservation scores are set with mean of 0 and one standard deviation equal to +1 or −1. CONSURF scores report similar results on a scale of 1 (least conserved) to 9 (most conserved). See the text and reference 23 for more details of this analysis.

We used a “marker-deflection assay” (12) to identify early beneficial mutants of a clone of B. cenocepacia strain HI2424 (wild type [WT]), initially isolated from soil (20) when growing in minimal media containing galactose as the sole carbon source. Equal fractions of otherwise identical Lac^+^ and Lac^–^ strains (the neutral markers) were inoculated in replicate tubes and propagated daily by 1:100 dilution for 6 days followed by 1:10,000 dilution for 6 additional days. Dilution levels were lower at the start to avoid extinction of the ancestral population, which had a long lag phase. If the initial 1:1 ratio of markers deviated to 3:1 in either direction (the deflection) or after 12 days of propagation (~102 generations), the experiment was stopped, and clones of both marker types were picked as putative “winner” and “loser” genotypes (Fig. 1A). These putative mutants were competed directly against the ancestral genotype of the opposite marker over 24 h in the same experimental conditions, and if they outcompeted the ancestor (relative fitness *r* > 0; see also Table S1 in the supplemental material), their genomes were analyzed by WGS to identify the causative adaptive mutations.

10.1128/mbio.00287-23.2 TABLE S1 Relative fitness of mutants captured during experimental evolution using marker deflection, assessed over 24 h versus WT HI2424. c.i., confidence interval. Download Table S1, PDF file, 0.03 MB. Copyright © 2023 Muraski et al. 2023 Muraski et al. https://creativecommons.org/licenses/by/4.0/ This content is distributed under the terms of the Creative Commons Attribution 4.0 International license.

In total, 19 putatively beneficial mutants were selected from three independent marker-deflection assays with the WT strain of HI2424 (see Table S2). Of these sequenced genotypes, eleven acquired single nucleotide mutations, seven had two to four mutations each, and two had no detectable mutation and were not considered further (see Table S2). Strong parallelism in the altered genes was evident: six genotypes acquired nonsynonymous mutations in *tilS*, encoding tRNA isoleucine lysidine synthetase (TilS); four genotypes were mutated in *ppc*, encoding phosphoenolpyruvate carboxylase; and a single nucleotide polymorphism (SNP) occurred in tRNA^Ile2^, the substrate of TilS. This report focuses on the mutations in *tilS* and tRNA^Ile2^ (Fig. 1B); a study of the *ppc* mutants will appear separately. Four of the six *tilS* mutants had only a single nucleotide substitution in that gene and no other, demonstrating that these mutations are the definitive genetic cause of the fitness advantage in GMM.

10.1128/mbio.00287-23.3 TABLE S2 (A) All mutations identified by WGS in 17 B. cenocepacia mutants selected for improved fitness in minimal medium containing galactose as sole carbon source. Clones 11 and 18 had no identifiable mutations and were excluded. Mutants in boldface are the focus of this study. (B) All mutations identified by WGS in 15 adaptive mutants of the B. cenocepacia “step 7” clone that had been preadapted in GMM under biofilm-selective conditions. Mutants in boldface are the focus of this study. Clones 1 and 15 had no identifiable mutations and were excluded. Download Table S2, PDF file, 0.07 MB. Copyright © 2023 Muraski et al. 2023 Muraski et al. https://creativecommons.org/licenses/by/4.0/ This content is distributed under the terms of the Creative Commons Attribution 4.0 International license.

We repeated this same design using a preadapted clone of B. cenocepacia that had been propagated in a similar medium but also selected for growth on plastic beads (21), again using marker-deflection versus an isogenic Lac^–^ mutant to capture new mutants. In total, 15 new mutants were isolated that included six with a single mutation and nine with two to four new mutations (see Table S2). Notably, four mutants acquired substitutions in tRNA^Ile2^ and nine acquired mutations in *ppc*, providing further evidence of genetic parallelism of adaptations. Because isolating the phenotypic contributions of these parallel mutations was complicated by the seven mutations this ancestor had previously acquired in an earlier experiment (21), we did not analyze these genotypes further. However, the parallel evolution of six nonsynonymous mutations in the essential *tilS* gene, as well as five mutations in its cognate tRNA^Ile2^ gene, supports the inference that selection acted to disrupt the interaction between these gene products. These genotypes became the experimental focus of this study.

To evaluate the evolutionary conservation of mutated TilS residues and whether they may be tolerant of variation, we aligned TilS amino acid sequences to the cocrystal structure of *Geobacillus kaustophilus* TilS and Bacillus subtilis tRNA^Ile2^ (PDB 3A2K) (22) using CONSURF (23). CONSURF computes site-specific rates of evolutionary divergence from phylogenetically representative homologous sequences using Bayesian inference; a static alignment for this PDB is available at the CONSURF web server (23). The positions of mutated residues are highly conserved (49 to 100%), and all are among the top 25% most conserved sites among 150 phylogenetically diverse homologs (Fig. 1C and D). Although mutations affected highly conserved positions, the parental BcTilS residue differs from the consensus residue in the multispecies alignment in some cases. For example, N274 aligns with the consensus R271; this residue is only Asn in 9.7% of the sequences. Likewise, N445K aligns with consensus S442 and is only Asn in 3.0% of the sequences. Of the six TilS mutants identified in the WT background (R208C, N274Y, P421L, N445K, and A244T found twice), all but R208C occurred alone, meaning that these single substitutions are the sole cause of the phenotypes reported here. Because R208C was not isogenic, this mutant was not studied further, although notably this residue is strictly (100%) conserved.

### Mutations in *tilS* and *tRNA^Ile2^* enhance growth in the absence of certain amino acids.

**FIG 2:**
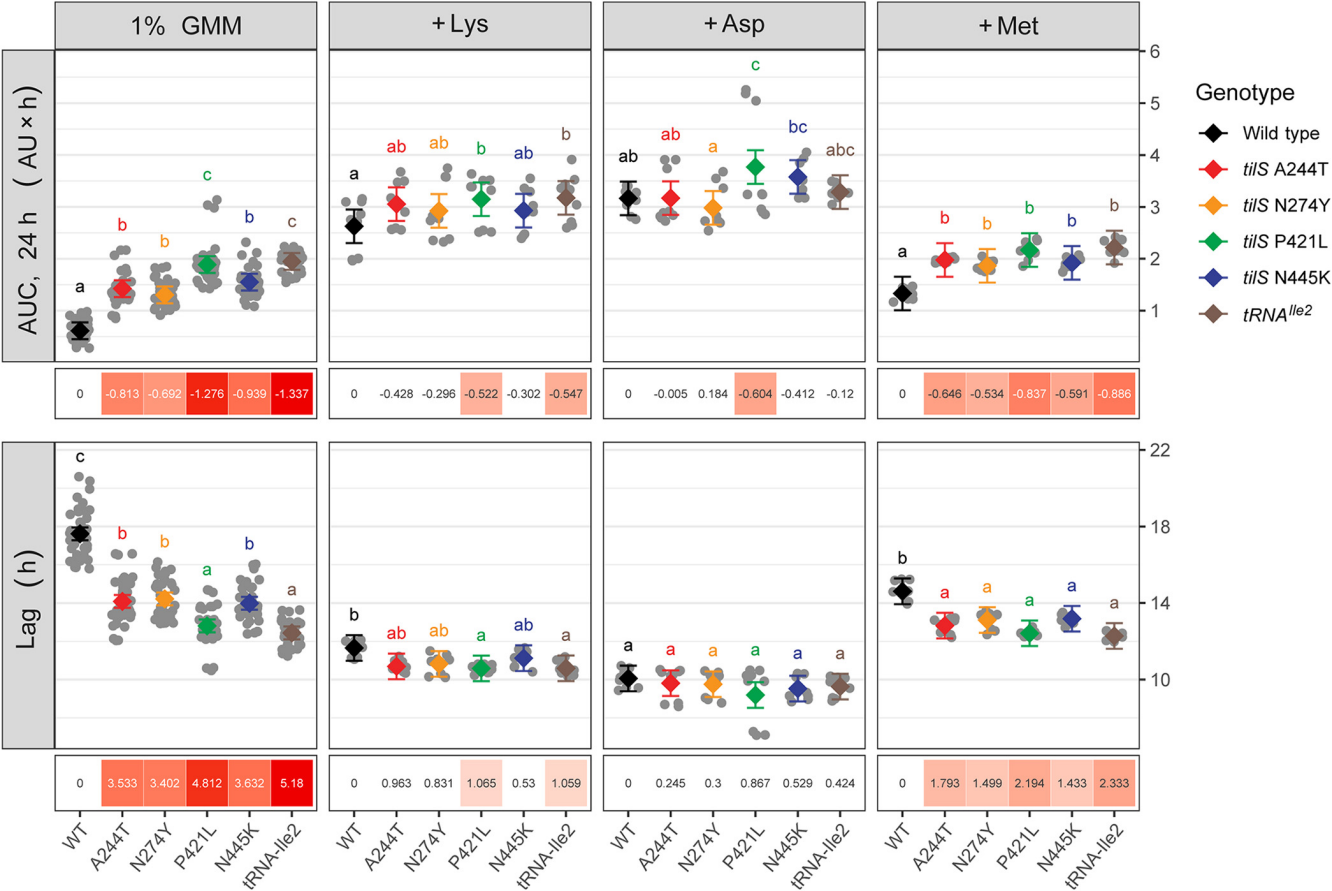
Fitness components of WT and *tilS* or *tRNA^Ile2^* mutants of B. cenocepacia. Increased fitness of *tilS* mutants (area under the curve, AUC, top row) results from reduced lag phase (Lag, bottom row) in GMM, but the fitness deficit of wild-type (WT) is complemented by added lysine (Lys) or aspartate (Asp) but not methionine (Met). Means and confidence intervals are indicated in black (WT) or color (mutants), with individual observations in gray. Letters above each data set denote results of *post hoc* pairwise means testing following two-way analysis of variance (ANOVA; *P* < 0.05) and corrected for multiple comparisons. Groupings are statistically indistinguishable if they share the same letter, but different groupings if letters differ. Quantitative differences in fitness measures (WT-mutant) are shown in boxes at bottom, and significant differences are shaded by magnitude of difference. AUC, area under the curve; AU, absorbance units at OD_600_.

We hypothesized that the parallel evolution of mutations in *tilS* and its cognate tRNA^Ile2^ resulted from selection to remedy some metabolic inefficiency tied to amino acid availability, given that lysine is a substrate of TilS. We therefore compared growth kinetics of *tilS* and tRNA^Ile2^ genotypes with WT in GMM in which they originally evolved and in GMM containing various nutritional supplements. As expected, fitness, defined as the area under the growth curve (AUC), was significantly greater for *tilS* and tRNA^Ile2^ mutants in GMM (Fig. 2). It is notable that fitness advantages of all mutants were statistically similar in most assays, suggesting that either disrupting the enzyme (TilS) or the substrate (tRNA^Ile2^) produce the same phenotype affecting growth (see Fig. S1). Supplementation with lysine or with aspartate, arginine, or isoleucine, each of which can be efficiently converted to lysine, complemented the fitness defect of WT and eliminated significant differences between genotypes (Fig. 2; see also Fig. S1). Consistent with this model that lysine deficiency favored these mutants, supplementation with amino acids that are metabolically distant from lysine (methionine and tryptophan) had no effect on fitness difference, although phenylalanine, which is not tied to lysine pathways, did complement WT fitness and improved growth of all mutants (Fig. 2; see also Fig. S1). The results suggest that scarcity of certain free amino acids, including lysine contributed to selection of the mutants.

10.1128/mbio.00287-23.6 FIG S1 Effects of supplements to GMM (column 1) on components of fitness that differentiate *tilS* mutants from the WT. AUC, area under the curve, for 24 or 36 h. Lag, carrying capacity (carry cap), and maximum growth rate (*V*_max_) are inferred from the growth curve (see Materials and Methods). Means and confidence intervals are in black (WT) and colored (mutants), with individual observations in gray. Letters above each dataset denote results of *post hoc* Šidák-corrected pairwise means testing with a cutoff of 0.05 following two-way ANOVA (*P* < 0.05). Groupings are statistically indistinguishable if they share the same letter, but different groupings if the letters differ. Quantitative differences in fitness measures (WT-mutant) are shown in boxes, and significant differences are shaded by magnitude of difference. AU, absorbance units at OD_600_; h, hours; Lys, lysine; Asp, aspartate; Met, methionine. Download FIG S1, PDF file, 0.7 MB. Copyright © 2023 Muraski et al. 2023 Muraski et al. https://creativecommons.org/licenses/by/4.0/ This content is distributed under the terms of the Creative Commons Attribution 4.0 International license.

**FIG 3:**
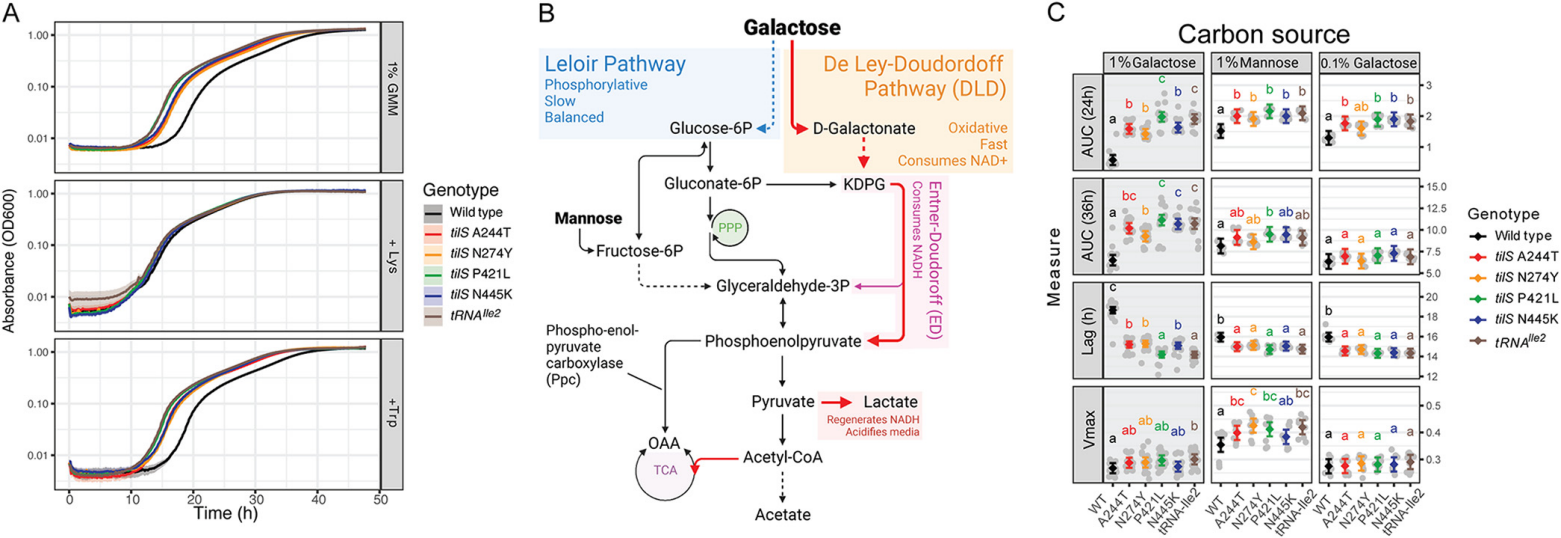
Fitness advantage of *tilS* and *tRNA^Ile2^* mutants is specific to imbalanced oxidative growth on substrates like galactose and in the absence of amino acids like lysine. (A) Lysine supplementation eliminates delayed lag of B. cenocepacia WT relative to *tilS* and *tRNA^Ile2^* mutants. Added lysine (middle panel), but not tryptophan (bottom) to the selection media (unaltered, top) complements the WT fitness defect relative to *tilS* mutants by reducing lag phase duration. Several other substrates eliminate fitness differences between WT and mutants (see Fig. S1). (B) Identity and concentration of the carbon source dictates relative fitness advantage of *tilS* mutants. Galactose can be metabolized via either the slower, redox-balanced Leloir pathway, or the faster combination of De Ley-Doudoroff (DLD) and Entner-Doudoroff (ED) pathways that causes redox imbalance and media acidification. Note that phosphoenolpyruvate carboxylase (Ppc) provides a shunt past pyruvate and avoids lactate production. (C) Fitness differences are greatest in 1% galactose (Gal), followed by 0.1% galactose, and least in 1% mannose (Man). Means and confidence intervals are in black (WT) and color (mutants), with individual observations in gray. Different letters distinguish statistically significant groupings determined by pairwise means testing after two-way ANOVA (*P* < 0.05). The gray panel background denotes GMM selective medium. AUC, area under the curve. Lag and maximum growth rate (*V*_max_) inferred from growth curves as described in Materials and Methods. AU, absorbance units at OD_600_.

Fitness benefits of mutants were further amplified over 36 h of incubation relative to the 24-h interval of serial transfer in the selection experiment. The growth curve dynamics (Fig. 3A) indicate why: cultures did not reach stationary phase until at least 36 h. These dynamics also show that fitness benefits of mutations arise mostly from the ability to emerge from lag phase ~3 h earlier than WT (Fig. 3A). On the contrary, the maximum growth rates of the mutants are indistinguishable from their ancestor regardless of supplementation (see Fig. S1).

### Fitness is dependent on carbon source and concentration.

The evolved mutants were isolated in selective minimal media with excess galactose as the only available carbon source. To test whether their fitness advantages were specific to this carbon source or its concentration, we substituted galactose with mannose or varied sugar concentrations. Mannose enters metabolism through the Leloir pathway via a fructose intermediate, whereas galactose enters through either the Leloir or the De Ley-Doudoroff (DLD) pathway, followed by the redox-imbalanced Entner-Doudoroff (ED) pathway (Fig. 3B). Given the high galactose concentration, we hypothesized that this sugar was predominantly metabolized through the DLD pathway via a galactonate intermediate, as the Leloir pathway is quickly overwhelmed (24). As predicted, substituting mannose for galactose greatly reduced the fitness advantage of the mutants (Fig. 3C). Furthermore, when galactose concentration was reduced from 1 to 0.1%, the growth curves more closely resembled that in 1% mannose. These results suggest that mutant fitness advantages depend on both amino acid scarcity and the primary pathway for substrate uptake and metabolism. Following research demonstrating that rapid growth on glucose by *Burkholderia* also relies upon the DLD-ED pathways (24), we also measured fitness in both 1 and 0.1% glucose. As predicted, all mutants grew earlier and to a higher density than WT in both concentrations, but with a greater advantage at higher glucose levels (see Fig. S2B). Further evidence of the prevailing use of DLD-ED pathway over the Leloir pathway was observed in measures of global transcription (see below), which indicated greater expression of gluconate kinase (*kgk*) than glucokinase (*glk*) across all genotypes studied here. These genes are the first committed steps of these pathways (paired *t* test, *t* = 4.51, df = 7, *P* = 0.0028).

10.1128/mbio.00287-23.7 FIG S2 Acidification of media by B. cenocepacia growth as a function of genotype and carbon source. (A) Representative *tilS* mutant N274Y (orange) reaches higher OD than WT (black) B. cenocepacia by the 24-h transfer period (dotted line) in selective RKS medium and in better buffered CSH medium, while acidifying the medium significantly more consequently. Lines and ribbons indicate means and standard errors by time point. (B) Relationship between growth, as measured by absorbance, and pH, in different media. The dotted line indicates the predicted buffering capacity of the medium. Download FIG S2, PDF file, 0.8 MB. Copyright © 2023 Muraski et al. 2023 Muraski et al. https://creativecommons.org/licenses/by/4.0/ This content is distributed under the terms of the Creative Commons Attribution 4.0 International license.

Another predicted outcome of galactose catabolism in *Burkholderia* is acidification of the media via the ED pathway, which induces secretion of organic acids as a by-product to restore redox balance (24). Rapid growth on galactose and flux through the ED pathway causes increased production of glyceraldehyde-3-phosphate and pyruvate, both of which consume NADH (Fig. 3B). Pyruvate is subsequently converted into lactate, some of which is secreted and causes media acidification. In prior experiments with this strain and growth media, we noticed acidification during growth but did not recognize this metabolic process (25). We confirmed the abundance of organic acids, including lactate, in the media following logarithmic growth by high-pressure liquid chromatography (HPLC). The fitness advantages of *tilS* and tRNA^Ile2^ mutants in GMM are largely explained by their reduced lag phase and hence earlier entry into exponential growth (Fig. 3A and C), but this change in growth could also affect the rate at which the medium acidifies. We measured the relationship between cell density and media pH for representative genotypes *tilS* N274Y and WT, in both the poorly buffered GMM media used in the selection experiment and in the standard M9 buffer (CSH) with 1% galactose (see Fig. S2A). As expected, N274Y grows faster than WT and reduces pH below 6.5 by 24h and below 6.0 at 36h. The WT strain eventually reaches similar OD600 and pH levels but approximately 4 h later, which translates into a large disadvantage in direct competition when the earlier-growing mutant competitor is acidifying the growth environment. On the contrary, growth in 1% mannose or 0.1% galactose does not acidify the media as quickly (see Fig. S2B). Because these growth curves were conducted in separate cultures, they cannot account for possible interactions between genotypes related to greater acid tolerance of mutants, suppression of the slower-growing WT, or metabolic cross-feeding. In summary, *tilS* mutants grow better on resources like galactose and glucose (see Fig. S2B) that are metabolized by the DLD and ED pathways, which causes redox imbalance and media acidification. Mutants acidify the medium faster, tolerate this stress, and indirectly limit growth of late-growing competitors, but they do not alter the relationship between growth and pH in these experimental conditions.

### Mutations in *tilS* greatly reduce lysidinylation of tRNA^Ile2^.

**FIG 4:**
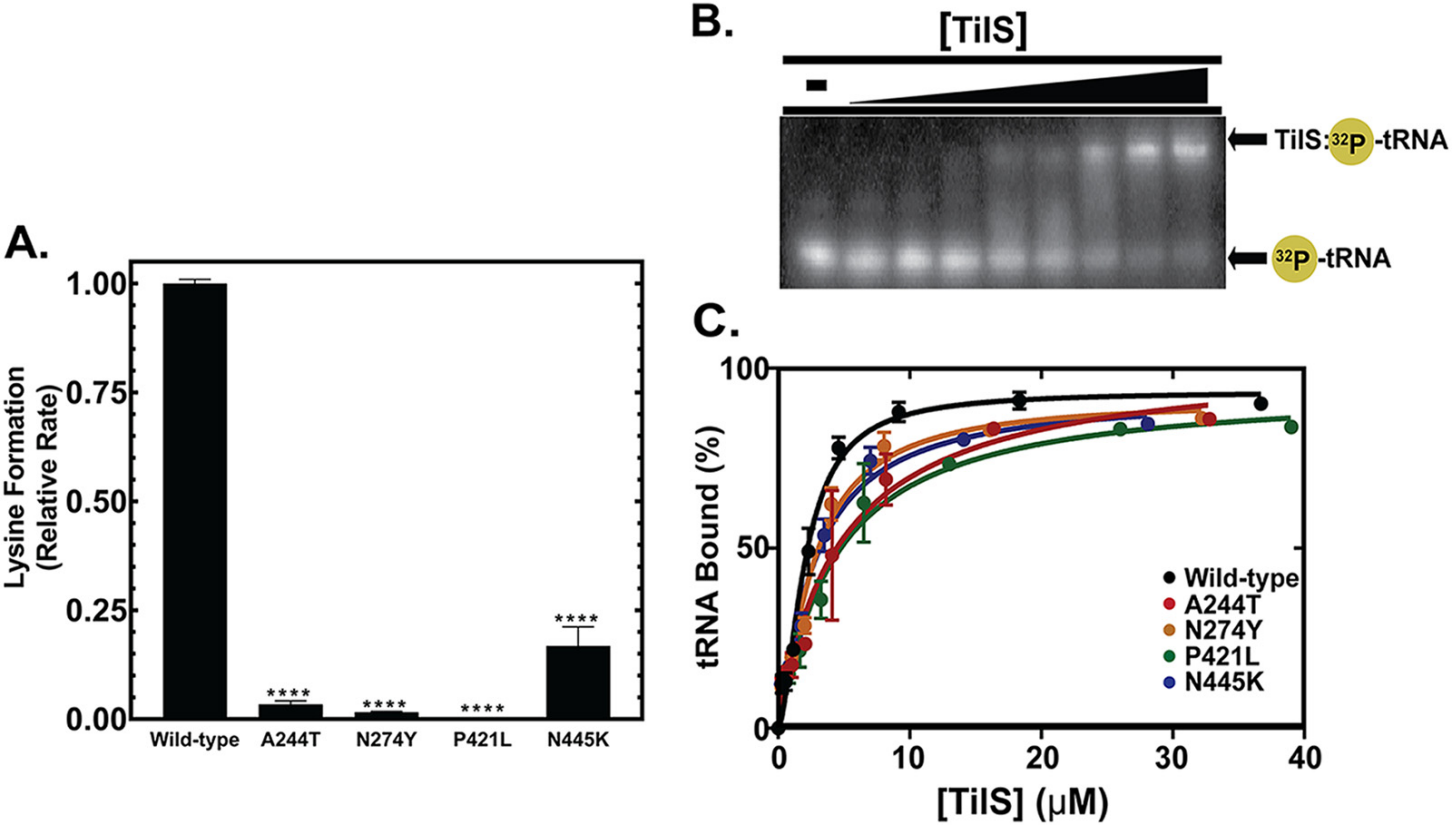
Evolved TilS mutations reduce catalysis significantly and tRNA binding modestly. (A) Catalytic activity of B. cenocepacia TilS variants. Lysidinylation activity was normalized to WT TilS; each protein variant was determined to be significantly (*P* < 0.05) different from the WT using one-way ANOVA. (B) Representative EMSA gel of TilS:tRNA^Ile2^ complex formation. ^32^P-radiolabeled tRNA^Ile2^ is separated from the ribonucleoprotein complex on a 10% native polyacrylamide gel. (C) Graphical summary of EMSA data fit to the Hill equation in Prism v7.0 (GraphPad).

The metabolic advantages of selected *tilS* and tRNA^Ile2^ mutations suggest that altered catalytic or binding functions of TilS with its cognate tRNA are responsible for B. cenocepacia fitness differences. The selected nonsynonymous mutations are distant from the TilS active site (Fig. 1B) but nonetheless alter a gene identified as essential for translation (16, 17, 26). We hypothesized that the mutations would impact TilS enzymatic function and tested this by measuring activity and structure of recombinant overexpression constructs of four TilS mutants in Escherichia coli. The influence of each mutation on TilS structure was assessed via circular dichroism. Only the N274Y variant displayed a modestly different α-helical content in its folded state compared to WT TilS (see Fig. S3), suggesting that most mutations had minimal effects on protein structure. [It should be noted that the N274Y variant also required lower IPTG induction to generate soluble protein.] We assayed each recombinant protein variant for its ability to catalyze lysidine synthesis using *in vitro* transcribed tRNA^Ile2^. Each mutation lowered catalytic activity compared to the WT enzyme, with A244T, N274Y, and P421L substitutions reducing activity by 30-fold to >100-fold (Fig. 4A) and N445K mutation reducing activity by 6-fold (see Table S3). Each selected mutation therefore caused loss of function *in vitro* despite affecting residues distal to the active site: residues 244 and 274 are ~25 Å away from the active site, while residues 421 and 445 are 60 and 75 Å away, respectively. This observation leaves open the possibility that the diminished activity of selected TilS mutants may be due to impaired tRNA binding.

10.1128/mbio.00287-23.4 TABLE S3 Rates of lysidinylation of each TilS mutant and WT. Errors represents the standard errors of the mean for each parameter, as determined from three replicates. NM, not measurable. Download Table S3, PDF file, 0.03 MB. Copyright © 2023 Muraski et al. 2023 Muraski et al. https://creativecommons.org/licenses/by/4.0/ This content is distributed under the terms of the Creative Commons Attribution 4.0 International license.

10.1128/mbio.00287-23.8 FIG S3 Circular dichroism of TilS variants. Spectra were collected in 20 mM Tris-HCl (pH 7.8) buffer. (A) Spectra represent the averages of experimental triplicates each of which were analyzed from technical triplicates. (B) Bar graph showing the determined α-helical content of each variant. Error bars indicate the standard deviations from three experimental replicates, and an asterisk (*) indicates a statistically different composition (*P* < 0.05) compared to WT TilS as determined using an ANOVA statistical comparison. Download FIG S3, PDF file, 0.07 MB. Copyright © 2023 Muraski et al. 2023 Muraski et al. https://creativecommons.org/licenses/by/4.0/ This content is distributed under the terms of the Creative Commons Attribution 4.0 International license.

### Evolved TilS mutations reduce but do not eliminate tRNA^Ile2^ binding.

To determine whether mutants affected the ability of TilS to complex with tRNA^Ile2^, we conducted electromobility shift assays (EMSAs) by incubating ^32^P-labeled tRNA^Ile2^ with increasing amounts of TilS variants. Mutants exhibited reduced binding affinity for tRNA^Ile2^ (Fig. 4C; see also Table S3) that likely contributes to the catalytic defect, but the <2-fold reduction in *K_d_* seems too small to explain the observed loss of enzymatic function approaching 2 orders of magnitude. These results suggest that reduced catalysis by TilS variants is not simply due to an inability to recognize tRNA^Ile2^. We next sought to determine whether mutants altered the *in vivo* pool of modified tRNA.

### tRNA^Ile2^ transcription levels are reduced in some mutants and diminish lysidine incorporation *in vivo*.

**FIG 5:**
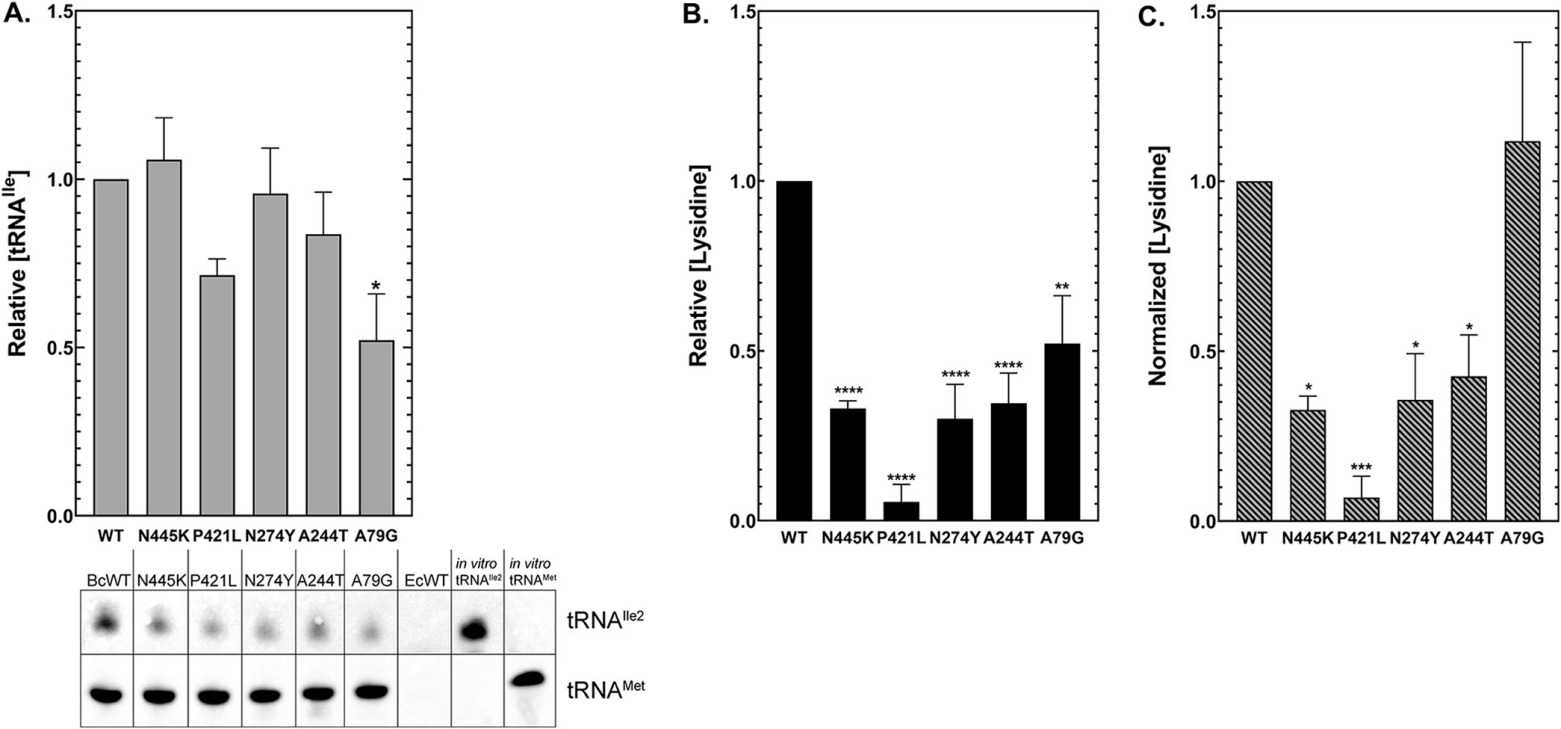
Cellular lysidine levels are decreased in TilS mutants but not in a tRNA^Ile2^ mutant. Total RNA was extracted from each strain, including tRNA^Ile2^A79G (A79G). (A) CY5-labeled probes specific for B. cenocepacia tRNA^Ile2^ or tRNA^Met^ were used to determine cellular tRNA abundance as a percentage of WT by Northern blotting. Total E. coli RNA was used as a negative control to demonstrate probe specificity; *in vitro*-transcribed B. cenocepacia tRNA^Ile2^ and tRNA^Met^ were used as positive controls. (B) Nuclease P1-digested total RNA was analyzed by LC-MS for the presence of lysidine. (C) Detected lysidine (see panel B) was normalized to tRNA^Ile2^ levels in each sample (see panel A) to adjust for differences in tRNA transcription levels between each sample and replicate by using ANOVA with *post hoc* testing for five biological replicates (*, *P* ≤ 0.05; **, *P* ≤ 0.01; ***, *P* ≤ 0.001; ****, *P* ≤ 0.0001).

One possible mechanism by which the cell can overcome diminished enzyme activity is by increasing synthesis of the enzyme’s substrate. We investigated whether the evolved mutants overproduced tRNA^Ile2^ *in vivo* by probing total B. cenocepacia RNA with CY5-labeled oligonucleotide DNAs targeting BctRNA^Ile2^ or BctRNA^Met^. The Northern blot analysis revealed no increase in tRNA^Ile2^ levels, but rather a modest decrease in the P421L mutant and an ~50% reduction in the A79G mutant (Fig. 5A). To test whether TilS mutants indeed failed to modify cellular tRNA^Ile2^, levels of individual nucleosides were analyzed by HPLC-coupled mass spectrometry. Lysidine modification is exclusive to tRNA^Ile2^, so loss of lysidine in the tRNA pool correlates to the loss of TilS activity. Lysidine levels in the TilS mutants mimicked the catalytic trends of these transcripts *in vitro* (Fig. 4A), with each of the mutant strains demonstrating reduced lysidine from the tRNA pool compared to the WT ancestor (Fig. 5C). Mutants A244T, N274Y, or N445K produced only ~30% of the lysidine level seen in the WT ancestor, while P421L produced only 10% of WT levels (see Table S3). Thus, the catalytic phenotypes observed *in vitro* are broadly consistent with those *in vivo*, and more importantly, increased fitness during growth in GMM is tied to the loss of the only known conserved function of the TilS enzyme.

### *tilS* mutations are broadly pleiotropic and their transcriptomes indicate enhanced metabolic efficiency.

**FIG 6:**
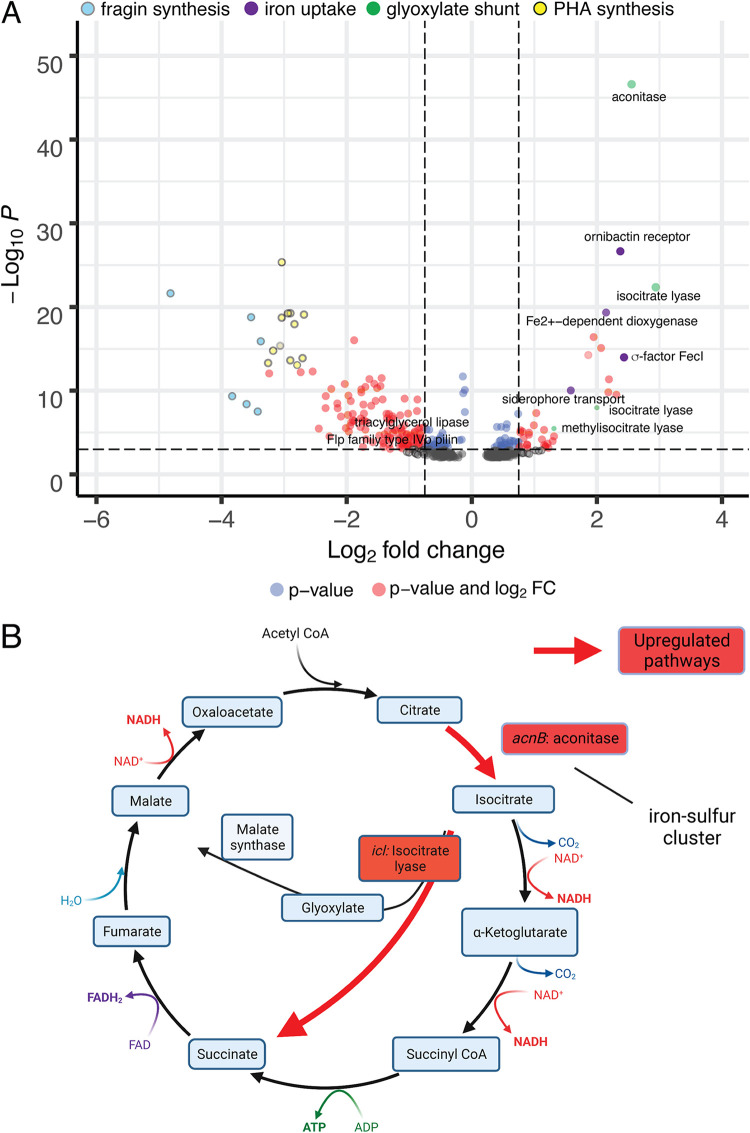
Genome-wide differences in expression between TilS N274Y and WT using RNA-Seq. (A) Volcano plot of 431 genes with fold change > 0.75 and/or *P* < 0.001 (denoted by dotted lines), with those meeting one or both criteria plotted in color. Most of the genes exhibiting the most significant changes in expression participate in four pathways, as shown. Comparisons between other mutants are shown in Fig. S4. (B) Shared, upregulated enzymes in *tilS* and *tRNA^Ile2^* mutants upregulate the glyoxylate bypass, likely to preserve carbon (acetyl-CoA) for gluconeogenesis.

We predicted that the large fitness gains of *tilS* and tRNA^Ile2^ mutants would associate with altered expression of gene sets that would suggest mechanisms of their growth advantage. Comparing mutant and WT transcriptomes was complicated by their different growth dynamics, rates of resource consumption, and media acidification (Fig. 3; see also Fig. S4). To evaluate genotype differences more independently of these environmental feedbacks, we isolated RNA from each replicate at equivalent optical density (but different time points) for transcriptome sequencing (RNA-Seq). Among hundreds of differently expressed genes, most outliers belong to four pathways (Fig. 6). First, each mutant showed upregulated iron uptake through Fe^3+^ siderophore receptors like ornibactin (*orbA*) and regulators like the FecI sigma factor (Bcen2424_1359), which are likely required to assemble iron-sulfur proteins that are in high demand during bacterial lag phase (27). Second, mutants upregulated the glyoxylate bypass through the iron-sulfur cluster enzyme aconitase (*acnB*), which was the transcript showing the greatest fold increases, and isocitrate lyase (*icl*), which preserves carbon for biomass generation (28). Two additional pathways are strongly suppressed in mutants: production of polyhydroxylalkanoates (PHAs), energy storage molecules that are typically produced in large quantities by *Burkholderia* but which consume acetyl coenzyme A (acetyl-CoA), and production of a newly discovered antifungal secondary metabolite called fragin, whose expression demands directly oppose that of *tilS* mutants (29). These differences are clearly illustrated in comparing N274Y with WT (Fig. 6) but are representative of other mutants, including *tRNA^Ile2^*, which shows similar effects on expression but differences of lower magnitude (see Fig. S4). Together, these data suggest that unmodified tRNA^Ile2^ or unoccupied TilS generate a signal or process that increases iron acquisition and conserves carbon as acetyl-CoA for synthesis of all cellular components from the sole substrate of galactose, while reducing investment in energy storage and metabolic by-products.

10.1128/mbio.00287-23.9 FIG S4 Volcano plots of comparisons between the transcriptomes of WT and *tilS* mutants A244T and P421L. Key similarities in strongly upregulated and downregulated genes with Fig. 6 (N274Y versus WT) are highlighted. Tables for these and other primary analyses are available elsewhere (http://github.com/vscooper/tilS). Download FIG S4, PDF file, 0.2 MB. Copyright © 2023 Muraski et al. 2023 Muraski et al. https://creativecommons.org/licenses/by/4.0/ This content is distributed under the terms of the Creative Commons Attribution 4.0 International license.

### *tilS* mutations are also selected in the long-term evolution experiment with *E. coli*.

**FIG 7:**
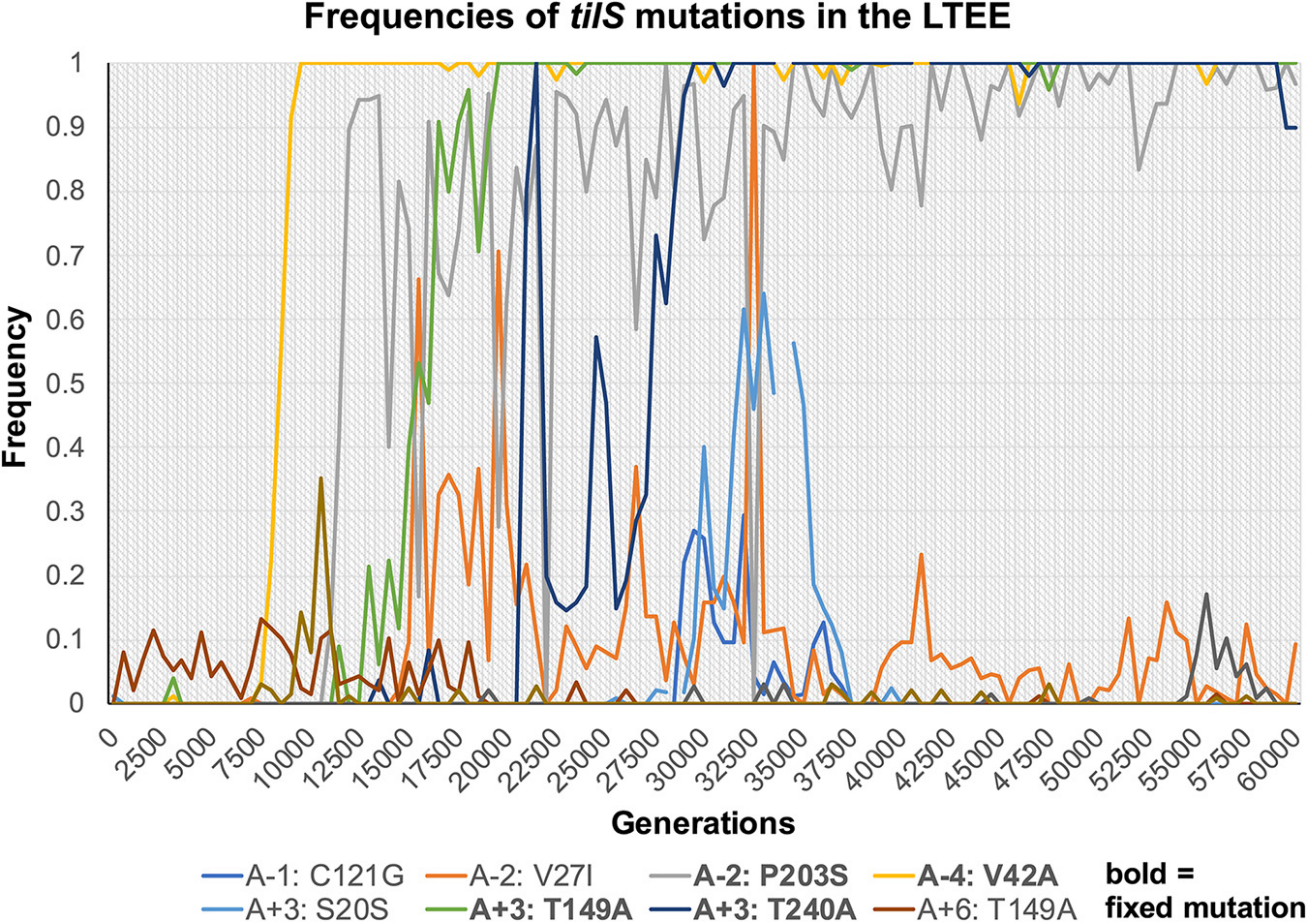
Frequencies of 10 *tilS* mutations in the LTEE that were detected at multiple time points during the experiment (30, 31). Three other mutations were only detected in single clones (see Table S4). Four mutations denoted in boldface reached 100% frequency: V42A in Ara–4, P203S in Ara–2, and T149A and T240A in Ara+3.

Our findings suggest that the enzymatic modification of tRNA^Ile2^ and/or a secondary function of TilS could experience selection in other evolution experiments with similar metabolic demands. The best studied and longest running evolution experiment is the long-term evolution experiment (LTEE), in which populations of E. coli have been propagated in minimal medium with glucose as sole carbon source for >70,000 generations. We hypothesized that this long-term selection for rapid growth and reduced lag phase on glucose could also select *tilS* mutants and searched published results of WGS of LTEE clones and populations for these mutations (30, 31). We identified 13 unique mutations (12 nonsynonymous) in five independent populations (see Table S4); four of these became fixed (100% frequency) in three different populations (Fig. 7). Based on the mutant activities shown here, we speculate that as many as three LTEE populations do not decode tRNA^Ile2^ as efficiently as the WT ancestral E. coli. This deficit could be significant because the E. coli genome encodes the AUA codon in the proteome in roughly 5 per 1,000 codons, which is far greater than the rate of 0.6 per 1,000 in B. cenocepacia. However, all fixed mutations arose in populations Ara–2, Ara–4, and Ara+3 that had evolved higher mutation rates, which increases the possibility that *tilS* mutations were not themselves beneficial but rather became fixed due to linkage with other more beneficial mutations (31). On the other hand, identical T149A mutations arose independently in two populations, ultimately fixing in one of them (see Table S4). This precise molecular parallelism indicates they are adaptive, but the enzymatic or fitness phenotypes associated with these mutations remain to be characterized.

10.1128/mbio.00287-23.5 TABLE S4 *tilS* mutations identified in a long-term evolution experiment with E. coli, as described by Good et al. (30). The dynamics of molecular evolution over 60,000 generations are depicted (30). Download Table S4, PDF file, 0.03 MB. Copyright © 2023 Muraski et al. 2023 Muraski et al. https://creativecommons.org/licenses/by/4.0/ This content is distributed under the terms of the Creative Commons Attribution 4.0 International license.

## DISCUSSION

Research into the genetic causes of adaptation is advancing rapidly with the aid of innovative high-throughput screens and efficient WGS. Often, beneficial genotypes converge on a few pathways or genes that were unexpected from the experimental environment and its stressors (2, 21). Selected mutants can reveal previously unknown genetic pathways to improved growth and disproportionately involve global regulators of expression (32, 33). These possibilities motivated our evolution experiments that included a reporter of selective sweeps to capture the earliest adaptive mutants in a simple laboratory environment medium with galactose as the sole carbon source. In two experiments with different B. cenocepacia strains, we identified 32 genetically distinct mutants associated with increased fitness, and remarkably 11 of them carried mutations in the TilS pathway, either in *tilS* gene itself (*n* = 7) or in its substrate *tRNA^Ile2^* gene (*n* = 4). All characterized *tilS* mutations reduced the known catalytic function of the TilS enzyme, the lysidinylation of the tRNA^Ile2^ anticodon that is essential for faithful and efficient translation of the AUA codon (18). Because prior research demonstrated that a partial knockdown of lysidine formation drastically hinders AUA decoding *in vivo* (15), our discovery of >10-fold reduced lysidine production by evolved mutants suggests that their translation of AUA codons is significantly impaired. However, these mutations improved fitness under our experimental conditions by reducing the lag phase prior to exponential growth (Fig. 2 and 3), suggesting that at least under certain conditions, sacrificing translational fidelity and efficiency can be beneficial for growth. Single SNPs in *tilS* also dramatically influence the global transcriptome (Fig. 6) and indicate that fitness gains are associated with increased usage of the glyoxylate bypass and iron scavenging and suppressed production of an antifungal secondary metabolite and PHA polymers. How mutated TilS or unmodified tRNA^Ile2^ alters the expression of these pathways and enables earlier exponential growth (Fig. 3) remains unclear, and we discuss some possibilities below. However, the convergent evolution of mutations in both *tilS* and *tRNA^Ile2^* suggests that interference with tRNA^Ile2^ lysidinylation was strongly favored.

Several studies have attempted to mutate or delete *tilS* from varied model organisms without success, except for the notable case of a *tilS* deletion mutant of B. subtilis that was suppressed by a co-occurring mutation in the anticodon of tRNA^Ile2^ from CAT to TAT (34). This experimental evidence that *tilS* is an essential gene is supported by its nearly universal conservation in bacterial genomes, save for a few that encode tRNA^Ile2^ with a TAT anticodon (35). However, the usage frequency of the AUA codon varies widely despite being relatively rare, from 0.6 AUA codons per 1,000 in G+C-rich genomes such as B. cenocepacia HI2424 to ~27 AUA codons per 1,000 in G+C-poor genomes such as Campylobacter species (36). It seems likely that varied demand for AUA decoding as a function of overall genome G+C content influences the essentiality of lysidinylation by TilS and could have facilitated the disruption of the TilS-tRNA^Ile2^ reaction in B. cenocepacia. Nonetheless, our discovery of 12 nonsynonymous mutations and 4 fixed mutations in the E. coli LTEE indicates that an organism with intermediate genome usage of AUA (5 per 1,000) can still tolerate mutations in *tilS* (see Table S4). Further, the dynamics of the 10 LTEE mutations that persisted at detectable frequencies over thousands of generations, combined with the fact that identical mutations arose in two independent lineages, suggest that mutated TilS was also advantageous during long-term selection for rapid growth in minimal glucose medium (Fig. 7). Together, the experimental selection of mutated *tilS* in two different systems demonstrates that partial loss-of-function mutations of this enzyme are not only tolerated, but even subject to positive selection under conditions of extreme metabolic flux that cause redox imbalance and demands for gluconeogenesis. Nevertheless, the fact that no deletion or premature stop mutations were selected in these experiments strongly indicates that translation of an intact TilS enzyme remains essential. The extreme pleiotropic effects of selected mutants on growth dynamics, resource usage, and global transcription point toward a yet-undiscovered moonlighting function for TilS, perhaps by binding other RNAs that regulate central metabolism and/or suppress production of secondary metabolites (Fig. 6).

We considered several explanations for the fitness advantages of *tilS* and *tRNA^Ile2^* mutations. First, we reasoned that genes enriched in rare AUA codons may have been selected for suppressed translation that an efficient TilS enzyme would otherwise enable. This form of attenuation by slowed translation speed has several precedents (37). However, the B. cenocepacia genome has only 20 clearly predicted genes with 4 or more AUA codons, and only ~1,100 AUA codons in the entire 7.8-Mbp genome distributed across ~800 genes (38) (see Text S1 in the supplemental material). Nonetheless, some of these 20 genes could play a role in modulating redox stress or metabolic regulation, such that their altered translation could influence fitness. Yet, no mutations or significant transcriptional differences were observed in this gene set, implying that any resulting differences would need to be studied by comparing protein levels. Specifically, we predict that AUA codons would specify misincorporation of Met instead of Ile in mutant genotypes more than typical background translation error, which we are currently measuring.

10.1128/mbio.00287-23.1 TEXT S1 Supplemental materials and methods. Download Text S1, PDF file, 0.1 MB. Copyright © 2023 Muraski et al. 2023 Muraski et al. https://creativecommons.org/licenses/by/4.0/ This content is distributed under the terms of the Creative Commons Attribution 4.0 International license.

Second, we considered that lysine levels became a limiting resource and their usage to modify tRNA^Ile2^ acted as a regulatory signal; however, infrequent this reaction. The fact that supplementation with lysine or other amino acids that are readily converted to lysine complemented the WT fitness deficit supports this explanation (Fig. 2). Further, mutant TilS enzymes exhibit reduced production of lysidine, and hence reduced consumption of lysine (Fig. 5). One possible source of lysine demand is the need to buffer an acidic cytosol or periplasm caused by organic acid by-products of growth using the ED pathway (Fig. 3) (24). In response to this acid stress, many microbes produce polyamines by decarboxylating amino acids like lysine and arginine into cadaverine, putrescine, or spermidine. These polyamines both directly buffer pH and interact with porins to prevent further proton uptake (39, 40), and research is mounting that they may have broader regulatory roles by activating the stringent response via the alarmone ppGpp (41). We tested this model by quantifying levels of amino acids in WT and mutant strains grown in our selective conditions by HPLC, and we also attempted to measure cadaverine. However, no significant differences in amino acid or polyamine levels were observed repeatably, suggesting that any interactions with polyamine production, should they exist, are transient and/or limited to the lag phase of growth distinguishing WT and mutants, where cell density may have been too low for these measurements.

Third, it is possible that TilS, in its role as a tRNA-modifying enzyme, binds other RNAs relevant for emergence from lag phase under these experimental conditions. Several major differences in mutant transcriptomes were identified that are not simple reflections of earlier exponential growth, given that we harvested cells at similar density and growth rate. Two major pathways that were upregulated seem to be the best candidates for such interactions (Fig. 6): (i) activation of the glyoxylate bypass to preserve carbon backbones from the tricarboxylic acid (TCA) cycle for biomass production or (ii) increased capacity for iron uptake that is a hallmark for entering exponential growth (42). Notably, the most upregulated gene in the TilS N274Y mutant is aconitase, which is an iron-sulfur cluster enzyme that leads to the glyoxylate bypass, as well as TCA. This change, combined with increased siderophore activity, suggests that mutant TilS may enable acquisition of trace metals to exit lag sooner. Future work will examine this potential secondary function of TilS. Fourth, it is possible that the modification state of tRNA^Ile2^ may act as a metabolic sensor in an unknown pathway. It is becoming appreciated that tRNAs can govern metabolism by linking their decoding roles with biosynthetic processes (37). Notable examples include the *miaB* gene that thiomethylates adenosine residues in tRNAs with NNA anticodons and also influences sporulation and antibiotic production in *Streptomyces* (43). In addition, the *miaE* gene that produces ms^2^i^6^A nucleoside modifications also influences aerobic growth on dicarboxylic acids in Salmonella (44). The most promising precedent may be a mutant of *tilS* in Helicobacter pylori that was associated with increased colonization of the mouse gut, an acidic environment, but this association was not explored further (45). In summary, based on the findings presented here, either the unmodified tRNA^Ile2^ or the unoccupied TilS enzyme likely play roles in regulating central metabolism that extend beyond their role in translational accuracy, but the identity of these genetic partners still elude us.

To conclude, a screen for beneficial genetic mutants of B. cenocepacia growing in a simple, inadequately buffered growth medium containing galactose as sole carbon source repeatedly selected mutants that should in theory fail to properly decode the rare AUA codon. These mutants improved growth by reducing the duration of lag phase, resulting in a large competitive advantage, and their advantages were specific to rapid, redox-imbalanced growth in medium lacking certain amino acids. TilS mutants also evolved in a long-running evolution experiment with E. coli in which exit from lag phase is also at a premium (46) and exhibited dynamics consistent with an adaptive role, together suggesting this tRNA modification pathway may influence fitness in diverse bacteria. The breadth of phenotypes altered by these mutations add to the growing evidence that the many modifications of tRNAs, each of which link to distinct pathways and essential building blocks, enable exquisite sensing of the cellular metabolic state.

## MATERIALS AND METHODS

A summary of our approach follows. More-detailed methods are available in Text S1 in the supplemental material.

### Bacterial strains and culture conditions.

B. cenocepacia HI2424, a soil isolate of a globally distributed clone causing infections in persons with cystic fibrosis and other immunological disorders, was the wild-type (WT) ancestor used in all experiments and is naive to laboratory conditions (20). A *lacZ*^+^ mutant was created by Tn7 insertion that is neutral for fitness but distinguishable on X-Gal plates (13). Selection experiments were conducted in a modified version of M9 minimal media with 1% galactose (GMM) added as the sole carbon source. Equal fractions of HI2424^lac^ and HI2424^lac–^ genotypes were added to 5 mL of GMM in test tubes grown on a roller drum and propagated by 1:100 dilutions for 6 days and 1:10,000 dilutions for up to 6 additional days. Every 72 h, a 10^−4^ to 10^−5^ dilution of each culture was counted on X-Gal (5-bromo-4-chloro-3-indolyl-β-d-galactopyranoside) plates to determine marker frequency; when it diverged beyond 3:1 or after 12 days (~120 generations), single clones were picked from both winning and losing marker types and compared to the ancestor for altered fitness. Clones found to be more fit (selection rate constant *r* > 0 [47] during 24 h of direct competition with the oppositely marked WT strain) were genotyped by WGS as described previously (5).

### Fitness assays.

Fitness was measured by growth curves in GMM mimicking selective conditions, except when supplements were added, e.g., 0.60 mM l-arginine, 0.10 mM l-aspartate, 0.40 mM l-lysine, 0.10 mM l-methionine, 0.20 mM l-phenylalanine, 0.05 mM l-tryptophan, or 0.18 mM iron(III) chloride hexahydrate; the modified M9 with 0.1% galactose or 1% mannose; or the standard M9 minimal medium recipe (48) with 1% galactose.

### TilS enzyme activity.

His-tagged WT and mutant TilS proteins were purified from E. coli expression vectors. Lysidinylation activity was monitored as previously described (22, 49). The efficiency of tRNA^Ile2^ binding was quantified by using EMSAs and Northern blotting. Cellular lysidine levels were determined by liquid chromatography-mass spectrometry (LC-MS) as described previously (50). RNA-Seq analysis of the mutants and WT was conducted as described previously (51), with the modifications described in Text S1.
