# Supplementary material for: Adaptation to Overflow Metabolism by Mutations That Impair tRNA Modification in Experimentally Evolved Bacteria
Source: mBio. 2023 Feb 28;14(2):e00287-23. doi: 10.1128/mbio.00287-23 (PMC10128029; doi:10.1128/mbio.00287-23)
Supplement: TABLE S1 [file mbio.00287-23-s0001.pdf]

Supplementary Table 1. Relative fitness of mutants captured during experimental evolution using marker deflection, assessed over 24h vs WT HI2424. Confidence interval = c.i.

Mutant	Relative Fitness *r*	95% c.i.
1	0.629	0.315
2	1.043	0.230
3	1.399	0.436
4	1.102	0.239
5	1.065	0.611
6	0.843	0.158
7	2.037	0.313
8	1.299	0.157
9	1.380	0.128
10	1.433	0.775
12	1.512	0.435
13	0.654	0.551
14	1.740	0.293
15	0.877	0.287
16	0.499	0.687
17	0.460	0.657
19	0.882	0.450
