# Supplementary material for: Adaptation to Overflow Metabolism by Mutations That Impair tRNA Modification in Experimentally Evolved Bacteria
Source: mBio. 2023 Feb 28;14(2):e00287-23. doi: 10.1128/mbio.00287-23 (PMC10128029; doi:10.1128/mbio.00287-23)
Supplement: TABLE S2 [file mbio.00287-23-s0002.pdf]

Supplementary Table 2. A. All mutations identified by WGS in 17 *B. cenocepacia* mutants selected for improved fitness in minimal medium containing galactose as sole carbon source. Clones 11 and 18 had no identifiable mutations and were excluded. Mutants in bold are the focus of this study. **B.** All mutations identified by WGS in 15 adaptive mutants of the *B. cenocepacia* "Step 7" clone that had been preadapted in GMM under biofilm-selective conditions. Mutants in bold are the focus of this study. Clones 1 and 15 had no identifiable mutations and were excluded.

Mutant	Locus	Annotation	Position	Amino Acid
**1**	**Bcen2424_2075 (*tilS*)**	**tRNA(Ile)-lysidine synthetase**	**2305668**	**R208C (CGC→TGC)**
1	Bcen2424_0661 (*eda*)	KDPG_aldolase	736233	P207L (CCC→CTC)
1	Bcen2424_3218	short-chain dehydrogenase/reductase SDR	59305	Q35H (CAG→CAT)
1	Bcen2424_4619	RND efflux system outer membrane lipoprotein	1597143	P267L (CCG→CTG)
2	Bcen2424_2426 (*ppc*)	phosphoenolpyruvate carboxylase	2691740	T41T (ACG→ACC)
2	Bcen2424_6531	acriflavine resistance protein	756381	V225A (GTC→GCC)
3	Bcen2424_2426	phosphoenolpyruvate carboxylase	2,691,756	D47N (GAC→AAC)
3	Bcen2424_5220	ATPase central domain-containing protein	2,277,234	L202P (CTC→CCC)
4	*hemC*	porphobilinogen deaminase	2,691,597	A7G (GCT→GGT)
**5**	***tilS***	**tRNA(Ile)-lysidine synthetase**	**2,304,955**	**N445K (AAT→AAG)**
**6**	***tilS***	**tRNA(Ile)-lysidine synthetase**	**2,305,028**	**P421L (CCG→CTG)**
7	Bcen2424_6711	major facilitator transporter	948,195	Δ3 bp (1110-1112/1329)
**8**	***tilS***	**tRNA(Ile)-lysidine synthetase**	**2,305,470**	**N274Y (AAC→TAC)**
9	Bcen2424_2627 (*pyk*)	pyruvate kinase	2911524	Δ1 bp (486/1437)
9	Bcen2424_0478	hypothetical protein	533982	D199E (GAC→GAG)
9	Bcen2424_4159	major facilitator transporter	1073395	L14P (CTC→CCC)
10	Bcen2424_2426	*ppc*: phosphoenolpyruvate carboxylase	2,691,740	T41T (ACG→ACT)
10	Bcen2424_0378 / *engB*	delta-aminolevulinic acid dehydratase/ribosome biogenesis GTP-binding protein YsxC	423,603	intergenic (-180/+73)
12	Bcen2424_0610 *(mpl)*	UDP-N-acetylmuramate	680,525	Q41* (CAG→TAG)
12	Bcen2424_4159 *(exuT)*	major facilitator transporter	1,073,395	L14P (CTC→CCC)
**13**	***tilS***	**tRNA(Ile)-lysidine synthetase**	**2,305,560**	**A244T (GCC→ACC)**

14	*ppc*	phosphoenolpyruvate carboxylase	2,691,740	T41T (ACG→ACT)
**15**	**Bcen2424_R0075**	**tRNA-Ile[2]**	**753458**	**noncoding: A79G**
16	Bcen2424_R0023	tRNA-Lys	900,484	noncoding: (TGGGAGGG)1→2 (62/77 nt)
17	Bcen2424_0524 / Bcen2424_0525	GTP-dependent nucleic acid-binding protein EngD/ubiquinone biosynthesis hydroxylase family protein	584933	intergenic (-119/-130)
17	Bcen2424_3118 (*queE)*	organic radical activating protein-like protein	3433117	I113V (ATC→GTC)
17	Bcen2424_5939	response regulator receiver modulated metal dependent phosphohydrolase	73960	A464A (GCA→GCC)
**19**	***tilS***	**tRNA(Ile)-lysidine synthetase**	**2,305,560**	**A244T (GCC→ACC)**

Supplementary Table 2B.

Mutant	Locus	Annotation	Position	Amino Acid
**2**	**Bcen2424_R0075**	**tRNA-Ile[2]**	**753394**	**noncoding: 15/79 nt**
3	*hemC*	porphobilinogen deaminase	2691276	P114Q (G->T)
4	*ppc*	phosphoenolpyruvate carboxylase	2691756	D47Y (G>T)
5	*ppc*	phosphoenolpyruvate carboxylase	2691756	D47Y (G>T)
5	Bcen2424_2427 (*phcB*) / Bcen2424_2428	SAM methyltransferase / XRE family transcriptional regulator	2695639	-105/-122 (C>G)
6	Bcen2424_0547	hypothetical	616106	Q28R (A>G)
6	Bcen2424_3441	hypothetical	271700	V178G (T>G)
6	*pyk*	pyruvate kinase	2911758	252/1437 (Δ1 bp)
7	Bcen2424_4077	enoyl-CoA hydratase	972475	A189G (C>G)
7	*ppc*	phosphoenolpyruvate carboxylase	2691740	T41T (G>C)
**8**	**Bcen2424_R0075**	**tRNA-Ile[2]**	**753442**	**noncoding: 63/79 nt (T>G)**
9	Bcen2424_5460 / Bcen2424_5461	Hsp90 ATPase 1	2559014	+108/-12 (G>A)
9	*ppc*	phosphoenolpyruvate carboxylase	2691630	G5R (G>A)
10	Bcen2424_4062	L-asparaginase II	956271	A202G (C>G)
10	Bcen2424_1349	periplasmic binding	1488451	D214E (C>A)
**10**	**Bcen2424_R0075**	**tRNA-Ile[2]**	**753442**	**Non-coding 63/79 nt (T>G)**
11	Bcen2424_4498	hypothetical protein	1450756	K455K (C>T)
11	Bcen2424_5069 / Bcen2424_5070	major facilitator transporter / porin	2093505	-145/+98 (C>G)
11	Bcen2424_0785	N-acetyltransferase	891452	A106P (G>C)

**11**	**Bcen2424_R0075**	**tRNA-Ile[2]**	**753442**	**Non-coding 63/79 nt (T>G)**
12	*ppc*	phosphoenolpyruvate carboxylase	2691630	G5R (G>C)
13	Bcen2424_5091	HlyD family protein	2121571	V325V (C>G)
13	*ppc*	phosphoenolpyruvate carboxylase	2691631	G5E (G>A)
13	Bcen2424_3038	urea amidolyase	3351696	A246P (G>C)
14	*ppc*	phosphoenolpyruvate carboxylase	2691625	S3C (C>G)
16	Bcen2424_2914	hypothetical protein	3218565	K93N (A>C)
16	*ppc*	phosphoenolpyruvate carboxylase	2691630	G5R (G>A)
17	Bcen2424_6582	MgtC/SapB	806881	A114A (T>C)
17	*ppc*	phosphoenolpyruvate carboxylase	2691757	D47V (A>T)
17	Bcen2424_3038	urea amidolyase	3351696	A246P (G>C)
