# Supplementary material for: Adaptation to Overflow Metabolism by Mutations That Impair tRNA Modification in Experimentally Evolved Bacteria
Source: mBio. 2023 Feb 28;14(2):e00287-23. doi: 10.1128/mbio.00287-23 (PMC10128029; doi:10.1128/mbio.00287-23)
Supplement: FIG S1 [file mbio.00287-23-s0005.pdf]

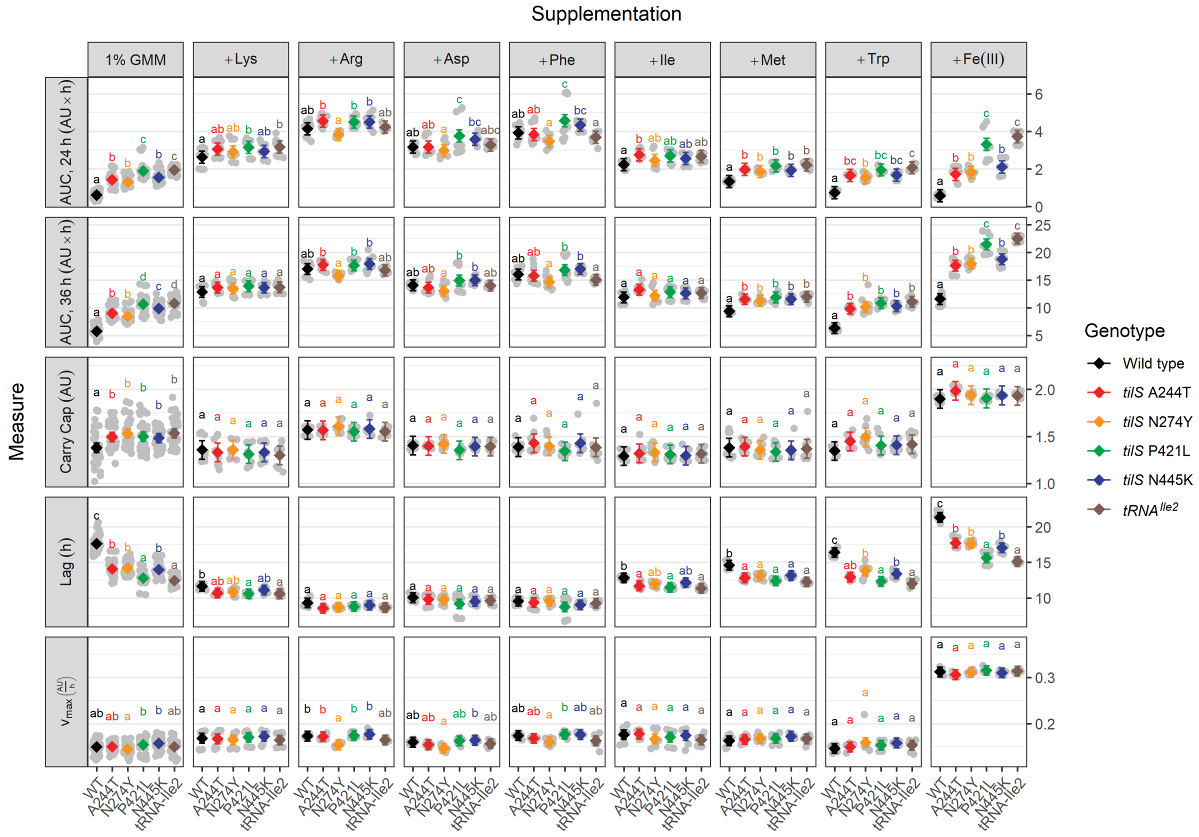

Supplementary Figure 1. Effects of supplements to GMM (column 1) on components of fitness that differentiate *tilS* mutants from WT. AUC = area under the curve, for 24h or 36. Lag, carrying capacity (carry cap) and maximum growth rate (v_{max}) are inferred from the growth curve (see methods). Means and confidence intervals are in black (WT) and colored (mutants), with individual observations in grey. Letters above each dataset denote results of post-hoc Šidák-corrected pairwise means testing with a cut-off of 0.05 following a two-way ANOVA, p<0.05. Groupings are statistically indistinguishable if they share the same letter, but different groupings if letters differ. Quantitative differences in fitness measures (WT-mutant) are shown in boxes, and significant differences are shaded by magnitude of difference. AU = absorbance units at OD600. h = hours. Lys = lysine. Asp = aspartate. Met = Methionine.
