# Supplementary material for: Adaptation to Overflow Metabolism by Mutations That Impair tRNA Modification in Experimentally Evolved Bacteria
Source: mBio. 2023 Feb 28;14(2):e00287-23. doi: 10.1128/mbio.00287-23 (PMC10128029; doi:10.1128/mbio.00287-23)
Supplement: FIG S2 [file mbio.00287-23-s0006.pdf]

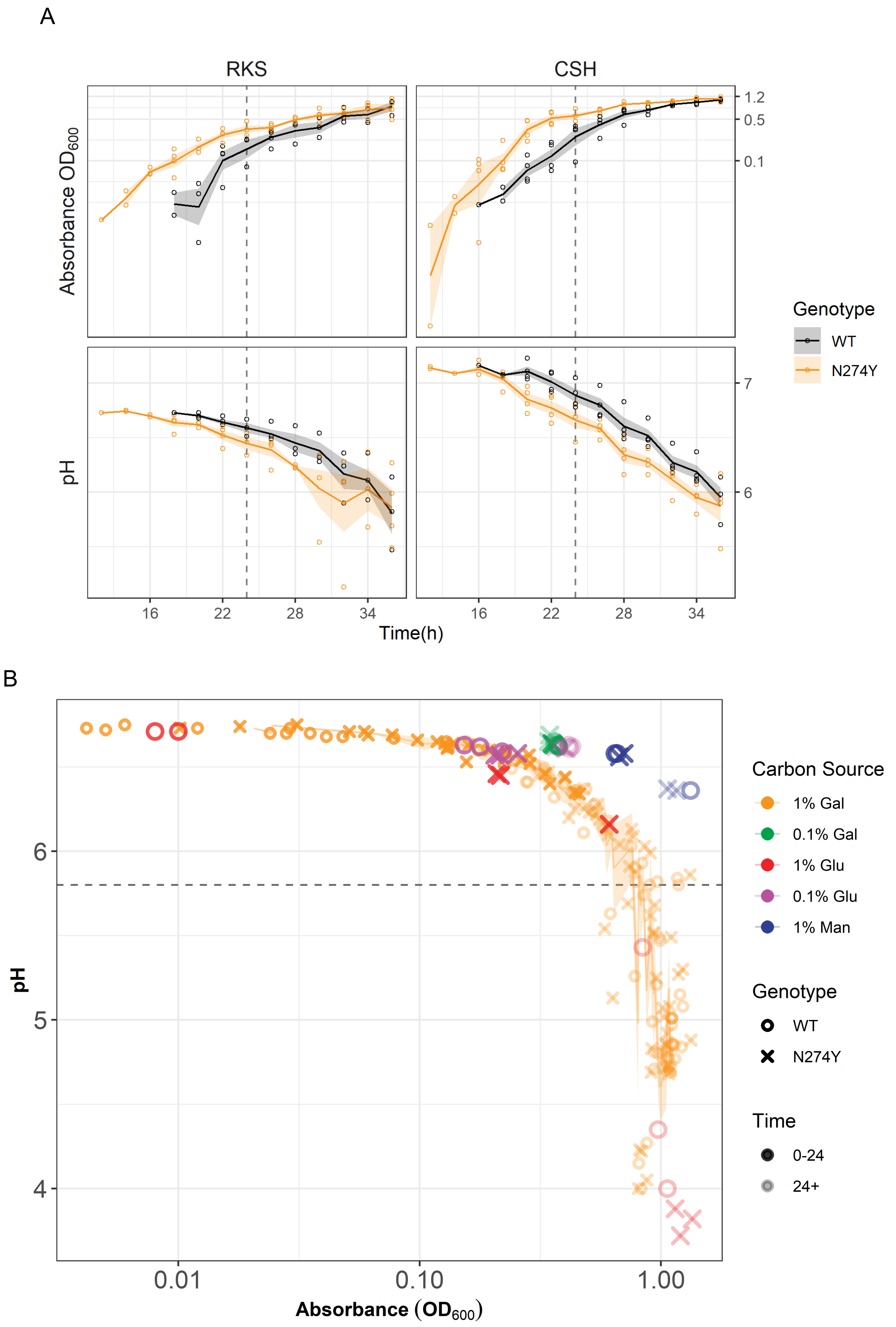
A
RKS
CSH
Absorbance OD600
pH
1.2
0.5
0.1
7
6
16
22
28
34
Time(h)
Genotype
WT
N274Y
B
6
5
4
pH
0.01
0.10
1.00
Absorbance (OD600)
Carbon Source
1% Gal
0.1% Gal
1% Glu
0.1% Glu
1% Man
Genotype
WT
N274Y
Time
0-24
24+

Supplementary Figure 2. Acidification of media by *B. cenocepacia* growth as a function of genotype and carbon source. A. Representative *tilS* mutant N274Y (orange) reaches higher OD than WT (black) *B. cenocepacia* by the 24H transfer period (dotted line) in selective RKS medium and in better buffered CSH medium, while acidifying the medium significantly more consequently. Lines and ribbons are means and standard error by time point. B. Relationship between growth, as measured by absorbance, and pH, in different media. Dotted line = predicted buffering capacity of the medium.
