# Supplementary material for: Adaptation to Overflow Metabolism by Mutations That Impair tRNA Modification in Experimentally Evolved Bacteria
Source: mBio. 2023 Feb 28;14(2):e00287-23. doi: 10.1128/mbio.00287-23 (PMC10128029; doi:10.1128/mbio.00287-23)
Supplement: TABLE S3 [file mbio.00287-23-s0003.pdf]

Supplementary Table 3. Rates of lysidnylation of each TilS mutant and WT. Error represents the standard error of the mean for each parameter as determined from three replicates. NM, not measurable.

	WT	A244T	N274Y	P421L	N445K
Lysidinylation rate (nM/min)	5.8 ± 0.8	0.17 ± 0.01	0.08 ± 0.01	NM	0.9 ± 0.1
Catalytic loss (fold)	1	34	72	>100	6
$tRNA^{Ile2}$ K_d (μM)	2.6 ± 0.3	4.6 ± 0.7	3.3 ± 0.4	4.5 ± 0.5	3.1 ± 0.3
Relative cellular lysidinylation	1	0.3 ± 0.09	0.3 ± 0.1	0.1 ± 0.05	0.3 ± 0.02
