# Supplementary material for: Adaptation to Overflow Metabolism by Mutations That Impair tRNA Modification in Experimentally Evolved Bacteria
Source: mBio. 2023 Feb 28;14(2):e00287-23. doi: 10.1128/mbio.00287-23 (PMC10128029; doi:10.1128/mbio.00287-23)
Supplement: FIG S3 [file mbio.00287-23-s0007.pdf]

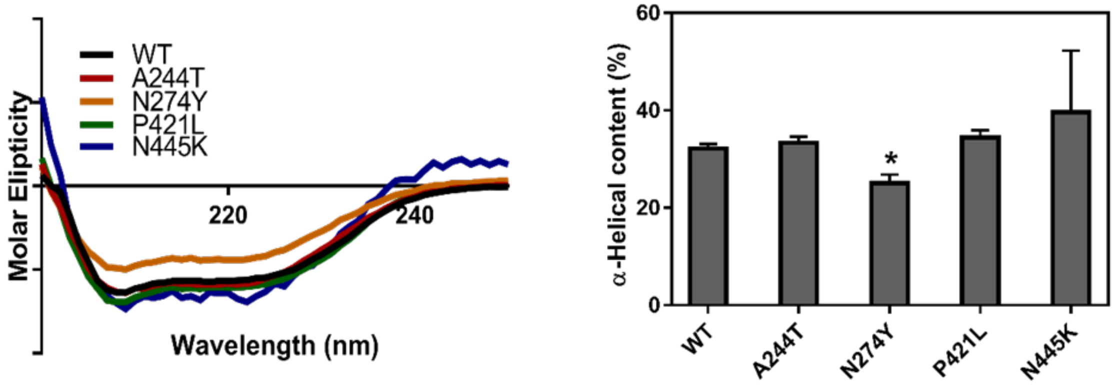

Supplementary Figure 3. Circular dichroism of TilS variants. Spectra were collected in 20 mM Tris-HCl (pH 7.8) buffer. A. Spectra represent the averages of experimental triplicates each of which were analyzed from technical triplicates. B. Bar graph shows the determined α-helical content of each variant; error bars indicate the standard deviation from three experimental replicates and * indicates statistically different composition ($P<0.05$) compared to wild-type TilS using an ANOVA statistical comparison.
