# Supplementary material for: Adaptation to Overflow Metabolism by Mutations That Impair tRNA Modification in Experimentally Evolved Bacteria
Source: mBio. 2023 Feb 28;14(2):e00287-23. doi: 10.1128/mbio.00287-23 (PMC10128029; doi:10.1128/mbio.00287-23)
Supplement: FIG S4 [file mbio.00287-23-s0008.pdf]

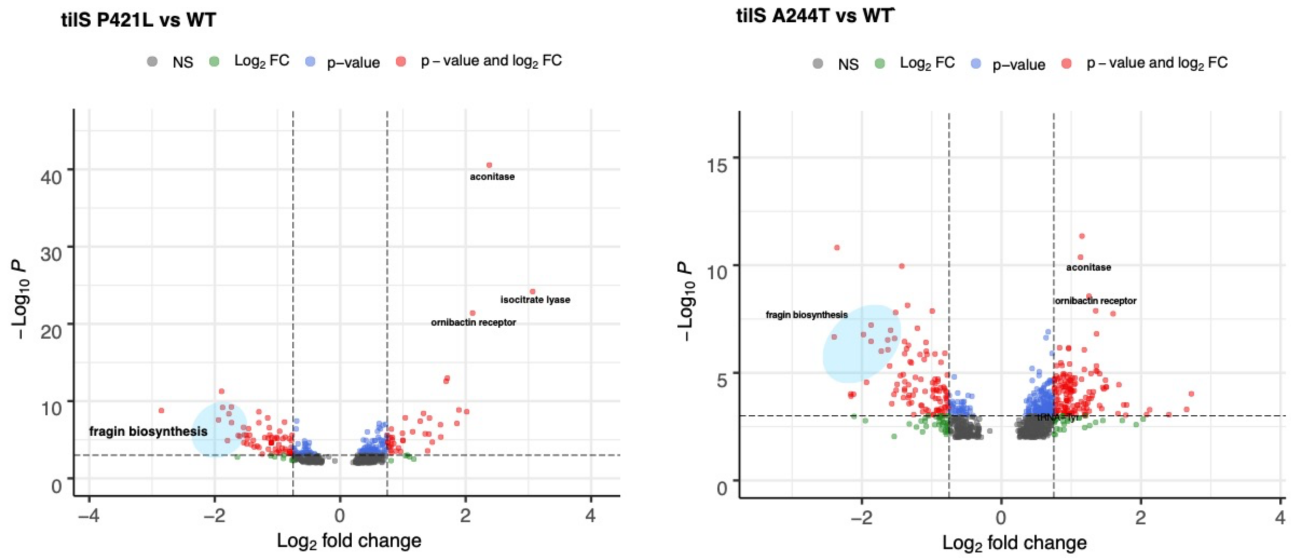

Supplementary Figure 4. Volcano plots of comparisons between the transcriptome of WT and *tilS* mutants A244T and P421L. Key similarities in strongly upregulated and downregulated genes with Figure 6 (N274Y vs WT) are highlighted. Tables of these and other primary analyses are available at http://github.com/vscooper/tilS
