# Supplementary material for: Adaptation to Overflow Metabolism by Mutations That Impair tRNA Modification in Experimentally Evolved Bacteria
Source: mBio. 2023 Feb 28;14(2):e00287-23. doi: 10.1128/mbio.00287-23 (PMC10128029; doi:10.1128/mbio.00287-23)
Supplement: TABLE S4 [file mbio.00287-23-s0004.pdf]

Supplementary Table 4. *tilS* mutations identified in the Long-Term Evolution Experiment with *E. coli*, as described in Good, BH. et al, The dynamics of molecular evolution over 60,000 generations. Nature 551:45–50.

Population	Gene position	Allele	Annotation	Maximum observed frequency
Ara-1	361	T->G	C121G	0.271
Ara-2	79	G->A	V27I	0.71
Ara-2	607	C->T	P203S	1
Ara-4	125	T->C	V42A	1
Ara-4	949	A->G	S317G	1 clone
Ara-4	101	A->G	Q34R	1 clone
Ara+3	60	C->T	S20S	0.61
Ara+3	220	C->A	P74T	1 clone
Ara+3	445	A->G	T149A	1
Ara+3	718	A->G	T240A	1
Ara+6	445	A->G	T149A	0.132
Ara+6	464	T->G	L155W	0.171
Ara+6	1106	T->G	V369G	0.352
